# Monitoring response to therapy in melanoma by quantifying circulating tumour DNA with droplet digital PCR for *BRAF* and *NRAS* mutations

**DOI:** 10.1038/srep11198

**Published:** 2015-06-22

**Authors:** Simon Chang-Hao Tsao, Jonathan Weiss, Christopher Hudson, Christopher Christophi, Jonathan Cebon, Andreas Behren, Alexander Dobrovic

**Affiliations:** 1 Olivia Newton-John Cancer Research Institute, Heidelberg, Victoria, Australia; 2 Department of Surgery, University of Melbourne, Austin Health. Heidelberg, Victoria, Australia; 3 Department of Pathology, University of Melbourne, Parkville, Victoria, Australia; 4 School of Cancer Medicine, La Trobe University, Bundoora, Victoria, Australia

## Abstract

We assessed the utility of droplet digital PCR (ddPCR) to evaluate the potential of using circulating tumour DNA (ctDNA) as a post therapy monitoring tool in melanoma by comparing it to serum LDH levels and RECIST scores. ddPCR was shown to be reliable in distinguishing mutant from wild type alleles with no false positives. Subsequently, we quantified ctDNA (^*V600E*^*BRAF*,^*V600K*^*BRAF* or ^*Q61H*^*NRAS*) in 6 stage IV melanoma patients across several time points during their treatment course. All tested patients had detectable ctDNA, which exhibited dynamic changes corresponding to the changes in their disease status. The ctDNA levels fell upon treatment response and rose with detectable disease progression. In our group of patients, ctDNA was more consistent and informative than LDH as a blood-based biomarker. In addition, *BRAF* mutant ctDNA as detected by ddPCR could be used diagnostically where the tumour block was unavailable. In conclusion, this study demonstrates the applicability of using ddPCR to detect and quantify ctDNA in the plasma of melanoma patients.

Melanoma incidence has been rapidly increasing worldwide^1^,^2^. Despite the considerable progress that has been made in the clinical treatment of melanoma with the introduction of targeted therapy and immunotherapy, reliable markers to predict treatment response or to measure early recurrent disease are still lacking.

The American Joint Committee on Cancer (AJCC) staging system is the only prognostic system widely accepted for melanoma. It considers the Breslow tumour thickness, presence of ulceration and the extent of nodal involvement for primary cutaneous disease^3^. For metastatic disease, it also considers the site of metastases and the serum lactate dehydrogenase (LDH) level. LDH is the only blood-based biomarker that has been incorporated in the staging system, as elevated levels of LDH are associated with higher disease burden and significantly decreased survival^3^. LDH also plays an important role as a stratification parameter in many clinical trials. However, LDH is non-specific and increases with many conditions and malignancies other than melanoma.

To assess if a patient is responding to treatment, the *response evaluation criteria in solid tumour* (RECIST) guideline is currently widely used. It helps clinicians to determine objectively whether the tumours have progressed (progressive disease (PD) with >20% increase in target lesion size), regressed (partial response (PR) with >30% decrease in target lesion size) or remained the same (stable disease (SD)) based on a set of radiological measurement criteria^4^. However, RECIST is subject to inter-scorer errors and radiological limitations such as insensitivity to small lesions (<10 mm), has significant costs, and involves considerable radiation exposure to patients. There is a clear need for markers that are more sensitive than RECIST, as easy to obtain as LDH, but better correlated to disease response.

One of the more recent marker types is circulating tumour DNA (ctDNA)^5^. ctDNA is released from tumour cells via various mechanisms including necrosis and apoptosis^6^,^7^,^8^. This enables real-time measurement of changes in tumour status as a result of therapy or recurrent disease. In breast and colorectal cancers, ctDNA has been reported to be significantly more sensitive for tracking disease status than traditional tumour markers^5^,^9^. Furthermore, it can also be used for predicting recurrence. Diehl *et al*. found all except one patient who had detectable ctDNA post colorectal cancer resection had tumour recurrence, whereas none of the patients who had undetectable post-surgical ctDNA levels developed recurrence^10^.

The detection of plasma ctDNA requires prior knowledge of tumour-specific mutations that can be used as tumour-specific markers. In melanoma, a handful of common mutations are present in up to 75% of tumours. The ^*V600E*^*BRAF* mutation is the single most common mutation in melanoma and is found in around 50% of cases^11^. Other common mutations are ^*V600K*^*BRAF* and several different *NRAS* mutations (e.g. *Q61R*, *Q61H* and *Q61K*) which occur mutually exclusively to the *BRAF* mutations^12^.

A recent publication from the BREAK-2 study, a phase II trial looking at the safety and clinical activity of the BRAF-inhibitor dabrafenib, explored the use of ctDNA as a predictor of outcome. The study used BEAMing, a digital technique that can sensitively detect small quantities of mutations in DNA, and found a positive correlation between baseline tumour burden and ^*V600E*^*BRAF* ctDNA levels. A higher baseline ^*V600E*^*BRAF* level was associated with higher baseline tumour burden, lower overall treatment response rate and lower progression free survival^13^. Another recent publication also used BEAMing to monitor response to therapy in a small group of melanoma patients^14^.

Here, we employed droplet digital PCR (ddPCR) technology, which can be readily used to quantify mutant DNA copies, with a detection sensitivity approaching 0.01%,^15^ to examine changes of the mutant ctDNA levels in melanoma patients across different time points. We considered that ddPCR for high frequency mutations would also enable ctDNA to be used as a sensitive and specific tumour marker. As ddPCR capable machines are increasingly available, this approach can be used to monitor treatment response in melanoma patients in an easy, non-invasive and cost-effective way.

## Results

### Cell line reconstruction studies

**Figure 1:**
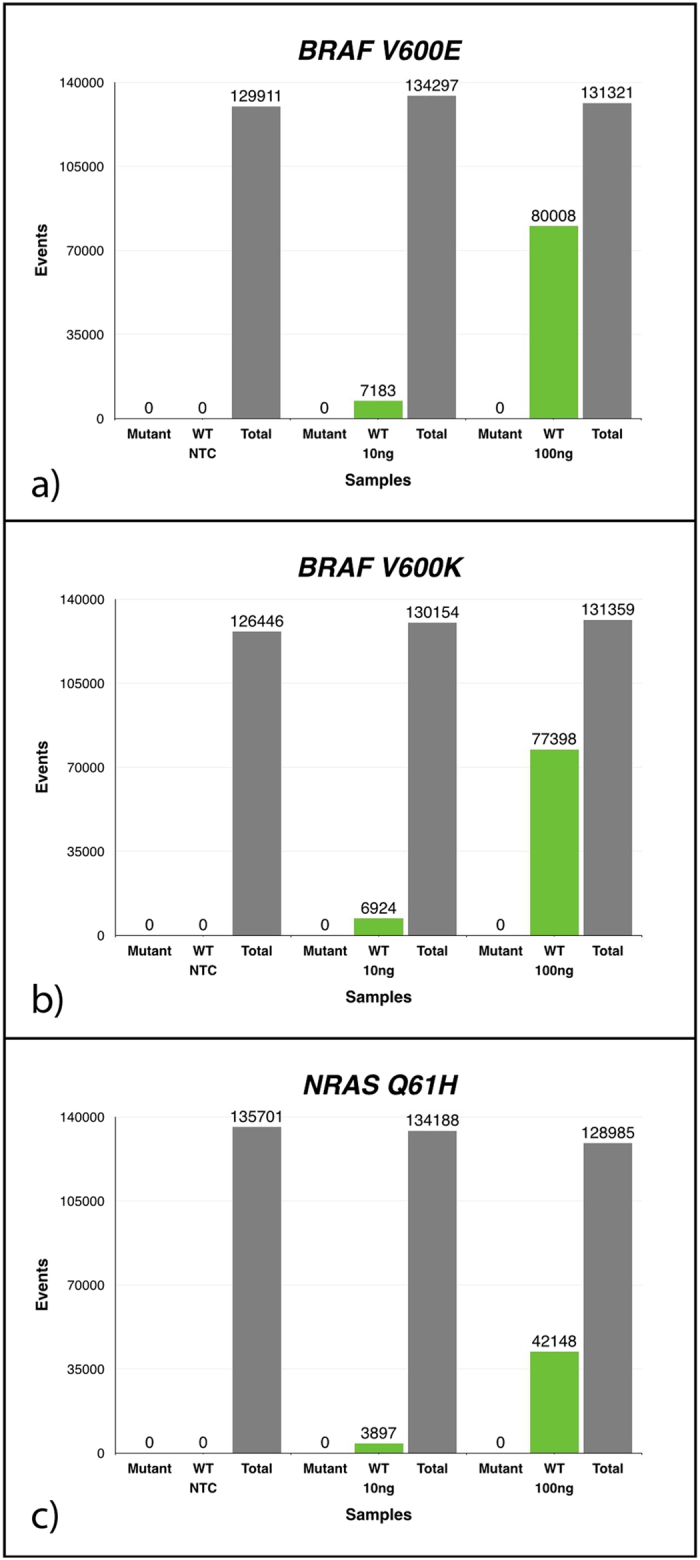
False positive determination for ^*V600E*^*BRAF*, ^*V600K*^*BRAF* and ^*Q61H*^*NRAS*. Graphs represent cumulative data for 8 repeats. Using known quantities of wild type (WT) DNA (nil, 10 ng and 100 ng of DNA) against both mutant and WT probes for (**a**) ^*V600E*^*BRAF*, (**b**) ^*V600K*^*BRAF* and (**c**) ^*Q61H*^*NRAS* mutations. There was no detectable mutant droplet count and the amount of droplets corresponding to WT DNA rose in proportion to the amount of DNA used. (NTC: no-template control. Total: total droplets generated.)

To examine the specificity of the primer-probe sets utilised, we tested these on genomic DNAs from melanoma cell lines with known mutational status^16^. The ddPCR system detected mutant DNA from all the cell lines with the same corresponding mutation. Furthermore, extensive replica testing of patients and cell lines DNA showed negligible differences between replicates when normalised to overall droplet counts (data not shown). Remarkably, there were also no false positive events detected using mutant probes on wild type plasma DNA (Fig. 1).

### Longitudinal Patient Studies

**Figure 2:**
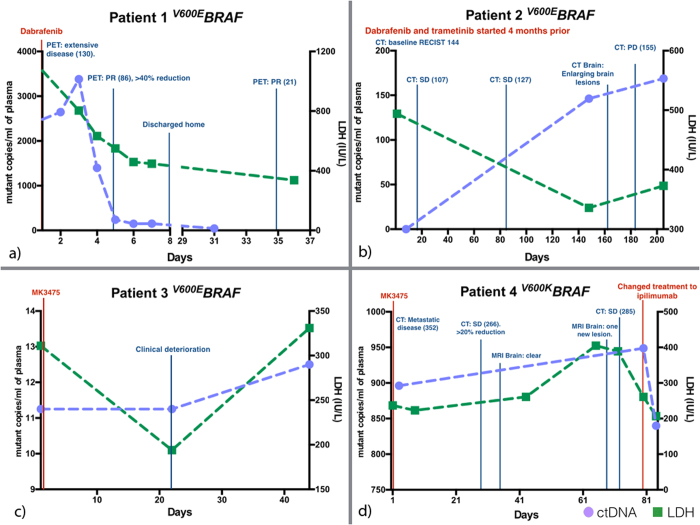
ctDNA and LDH level monitoring with clinical follow up for patient (a) 1, (b) 2, (c) 3 and (d) 4. ctDNA and LDH levels recorded with corresponding scan results. ctDNA level showed dynamic changes corresponding with disease progression. (Numbers in brackets correspond to the RECIST score. PR: partial response. PD: progressive disease. SD: stable disease.) The upper limit of normal LDH level in an adult is 250 IU/L. (**a**) Patient 1 showed a 98.3% decrease in ctDNA level following initiation of dabrafenib. The level stayed low as patient improved clinically with tumour shrinkage seen on PET scans with associated falling in RECIST score. (**b**) Patient 2 developed drug resistance evident with increasing tumour size. The ctDNA level also increased accordingly. However, patient’s LDH level fell paradoxically with rising tumour burden. (**c**) Patient 3 had multiple spinal metastases and we were unable to perform radiological scans to monitor the disease progression secondary to the metallic implant to stabilize the spine. The patient deteriorated despite new drug treatment (MK-3475). The LDH level fell initially after initiation of the new drug and then rose. The ctDNA level on the contrary never decreased with the new treatment. (**d**) Patient 4 started MK3475 after failing dabrafenib and trametinib with increasing intra and extra-cranial metastasis. All extracranial lesions responded well but the intracranial lesions continued to progress. This is reflected in the generally increasing ctDNA level, potentially representing remaining disease activity in treatment resistant intracranial lesions. The level fell when ipilimumab was added to the treatment regimen.

**Figure 3:**
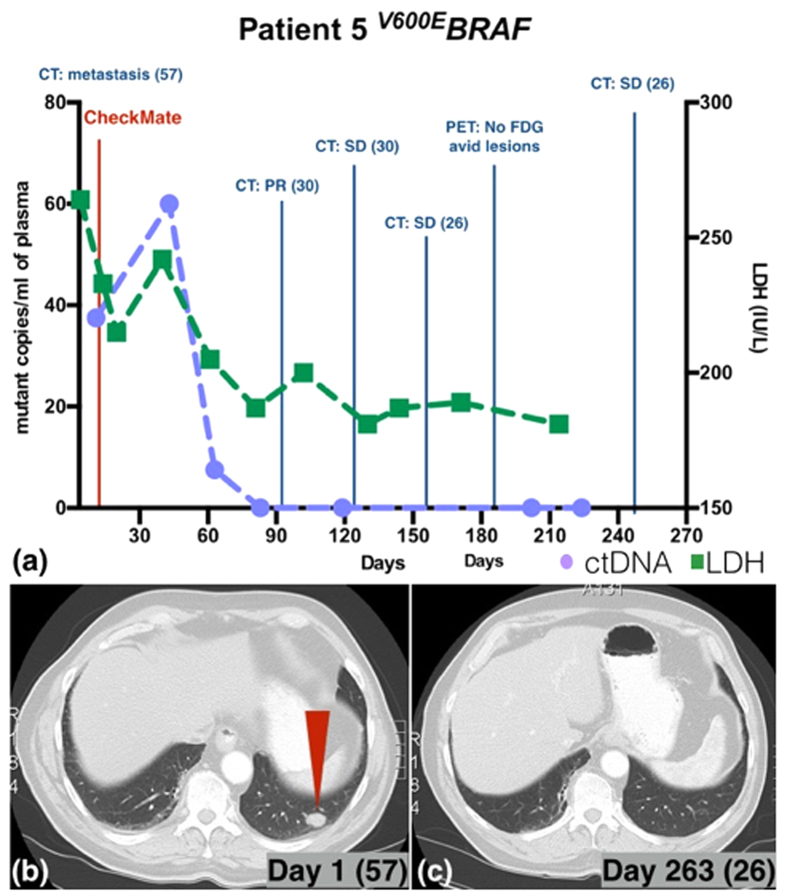
ctDNA and LDH level monitoring for patient 5 with corresponding radiological findings. (**a**) Patient 5’s ctDNA level fell following treatment (CheckMate 067 trial; anti-PD-1 antibody nivolumab alone versus anti-CTLA-4 antibody ipilimumab alone versus combination of the two). The CT scans also showed disease regression with (**b**) red arrow points to lung metastasis on day 1 of treatment and (**c**) the corresponding site on day 263 with no evidence of disease. PET scan performed on day 188 showed no metabolically active lesions (Fig. 3a).

**Figure 4:**
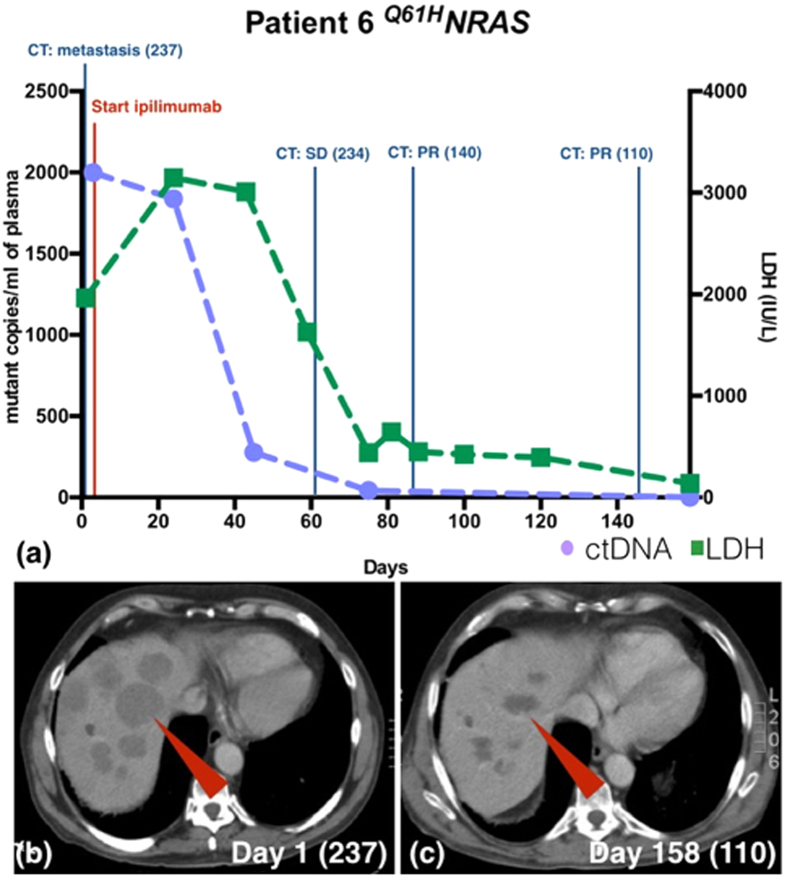
ctDNA and LDH level monitoring for patient 6 with corresponding radiological findings. (**a**) Patient 6’s ctDNA level fell rapidly after initiation of ipilimumab consistent with tumour shrinkage. The LDH level started to fall one month later than ctDNA level. (**b**) The CT image shows multiple large liver metastases (red arrow). The RECIST score was 237 on day 1 of treatment. (**c**) On day 158 since initiation of ipilimumab, the liver metastases have shrunken significantly (RECIST score 110).

**Figure 5:**
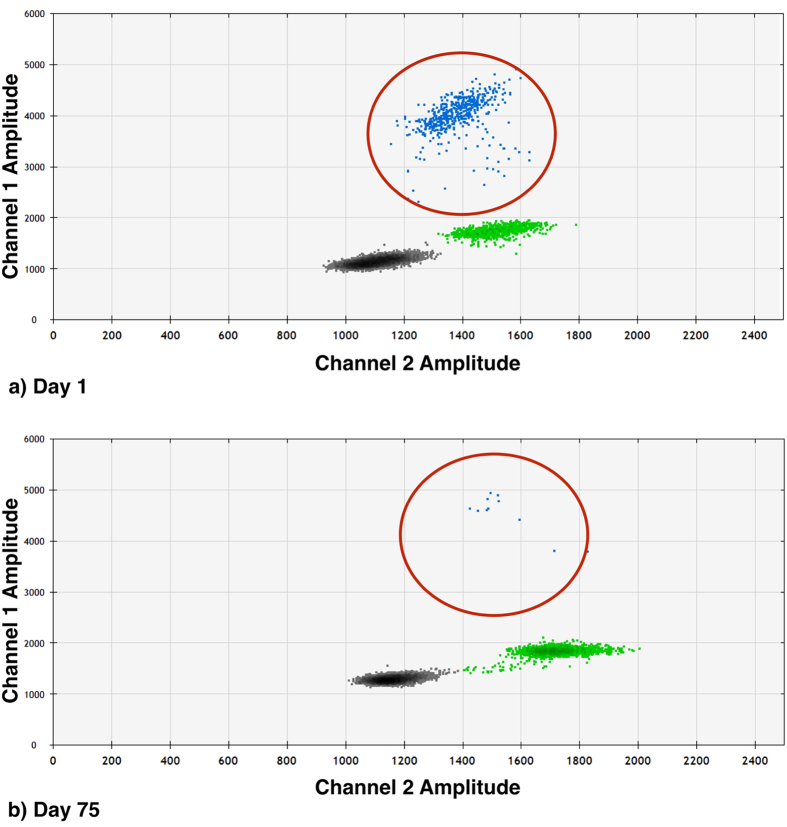
ddPCR results for patient 6. Following initiation of ipilimumab, patient 6 had significantly lower amounts of ^*Q61H*^*NRAS* in plasma. (Blue dots are ^*Q61H*^*NRAS*, green dots are wild type *NRAS*, and grey dots are droplets without DNA of interest.)

All patients with serial bloods tested (patients 1–6) had detectable ctDNA and showed changes corresponding to changes in their disease status (Figures 2–5 and table 1).

Patient 1 (^*V600E*^*BRAF*) was given dabrafenib for extensive metastatic disease. A PET scan performed 4 days after initiation of treatment showed greater than 40% reduction in fluorodeoxyglucose (FDG) uptake (uptake is associated with cellular metabolic activity) which decreased further one month later. The patient also showed significant clinical improvement progressing from being incapacitated by his disease to be able to go home with minimal supportive care required. The patient had a 98.3% fall in ctDNA level as his tumour responded to dabrafenib after one week of treatment. The LDH level on the other hand, although decreasing following treatment, remained well above the upper limit of normal, which is 250IU/L (Fig. 2a).

Patient 2 (^*V600E*^*BRAF*) was on dabrafenib and trametinib (a MEK-inhibitor) for 4 months prior to enrolment into this study. The patient had responded to treatment and presented with undetectable ctDNA at study commencement. The patient later developed drug resistance and progressive disease, which was diagnosed 9 months after initiation of treatment. This correlated with a rising RECIST scores and detectable ctDNA in plasma. In contrast, the patient’s LDH level fell initially despite a rising tumour burden (Fig. 2b).

Patient 3 (^*V600E*^*BRAF*) had multiple spinal metastases with several metal plates inserted for stabilization. This made radiological images difficult to interpret secondary to the artifacts and therefore tumour score with the RECIST criteria could not be obtained. The patient presented with progressive lower limb weakness whilst on dabrafenib and trametinib. The treatment was subsequently changed to MK3475 (a trial PD-1 inhibitory antibody) on compassionate basis but the patient continued to decline clinically. The patient’s LDH fell initially after starting the new therapy but ctDNA levels remained high. (Fig. 2c).

Patient 4 (^*V600K*^*BRAF*) started MK3475 when he developed progressive disease while taking dabrafenib and trametinib with increasing intra- and extracranial metastasis. All extracranial lesions responded well to MK-3475 (as known as pembrolizumab, a PD-1 inhibitor) as reflected by a falling RECIST score, but the intracranial lesions continued to progress. Both ctDNA and LDH levels continued to increase during this time. Treatment was subsequently changed to ipilimumab, which resulted in decline in ctDNA and LDH level (Fig. 2d).

Patient 5 (^*V600E*^*BRAF*) presented with extensive metastatic disease and was enrolled in the CheckMate 067 trial (anti-PD-1 antibody nivolumab alone versus anti-CTLA-4 antibody ipilimumab alone versus combination of the two). After an initial increase to 60 copies/ml from 37.5 copies/ml, ctDNA level fell rapidly to 0 copies/ml on day 83 and remained below the detection limit. (Fig. 3a). The LDH level followed the same general trend as ctDNA level. Follow up CT scans also demonstrated tumour shrinkage (Fig. 3a). The PET scan performed on day 188 showed no metabolically active lesions and the tumour seen on CT scans from day 1 of treatment (Fig. 3b red arrow) was resolved on day 263 (Fig 3c).

Patient 6 (^*Q61H*^*NRAS*) presented with extensive metastatic disease and was started on ipilimumab. The tumour responded well with significant reduction in tumour burden and falling in RECIST scores. The patient also had a rapid (86%) decline in ctDNA level by the 45^th^ day after therapy commencement, with LDH level starting to decline on the 59^th^ day. (Fig. 4a). The overall response is illustrated in fig. 4b with multiple large liver metastases, RECIST score 237 (red arrow) on day 1 of treatment compared to dramatic tumour shrinkage on day 158 with a RECIST score of 110 (Fig 4c). Figure 5 gives an example of the ddPCR raw data.

In addition, we studied a patient with a history of resected high-grade cutaneous melanoma who presented with metastatic disease presumed to be melanoma one year after initial diagnosis. The initial biopsy did not contain any viable tumour cells when examined by pathologists, and hence no mutation testing was done on the sample, rendering the patient ineligible for BRAF inhibitor based therapies. Upon examination of plasma-derived ctDNA, we found the ^*V600E*^*BRAF* mutation using the specific ddPCR assay. This opens the possibility of directly interrogating the plasma for actionable mutations whenever the primary tumour is unavailable. Such strategies will be particularly successful in diseases like melanoma where certain tumour-specific mutations are present at high frequency.

## Discussion

Our study validates the applicability of using ctDNA measured by ddPCR to assess the therapeutic response. Previously, Oxnard *et al*. showed that ddPCR for ^*V600E*^*BRAF* mutations could be used to track response to vemurafenib in melanoma patients^17^. In our case series, the ctDNA level as measured by ddPCR was a superior reflection of the treatment response and emergence of treatment resistance then LDH levels. While LDH levels often changed accordingly, they were slower in reflecting changes in disease status and were inaccurate in at least 2 of the patients tested (patient 2 and 3). This is consistent with findings by Bettegowda *et al*., and Dawson *et al*., where they found that ctDNA is significantly more accurate for tracking disease status than the traditional serum markers such as CEA and CA 15-3, for colorectal and breast cancer, respectively^5^,^9^.

Unfortunately, although BRAF-inhibitors and MEK-inhibitors can lead to rapid and dramatic treatment responses, phase III trials show that only a portion of the patients respond to the drugs and the survival benefit is limited as most patients will develop resistance within months of treatment^18^. With the newer immune modulating drugs such as PD-1 (MK-3475) and CTLA-4 (ipilimumab) blocking antibodies, durable responses can be seen in a proportion of patients^18^,^19^. However, as with the kinase inhibitors, a substantial proportion of patients will not respond to the treatment, or respond at later time-points after treatment initiation when assessed with standard RECIST^20^. Taking a “wait-and-see” approach may cost a patient valuable time suitable for alternative interventions. As seen in patient 6, ctDNA levels may predict responses to immuneinterventions long before responses are reflected in LDH levels and potentially CT/PET scans. This emphasises the necessity and advantage of having an easily performed sensitive method to frequently monitor treatment response. The rapid change in ctDNA levels opens the possibility to detect treatment response and emergence of resistance early and may allow for an alternative treatment to be introduced before major declines in health parameters, and at a time-point where therapeutic success is still achievable.

Current radiological definition of disease progression based on RECIST requires a more than 20% increase in measurable tumour size. The time to reach this threshold can take up to several months, which can result in prolonged, unnecessary drug toxicity and spent on health care resources. LDH is the only blood-based biomarker that has been incorporated in the management of melanoma patients where an elevated level is associated with higher disease burden and decreased survival^3^. However, LDH is neither sensitive nor specific, and it has been shown to be an unreliable marker for monitoring treatment response, as demonstrated in our study^21^.

Similar results have been obtained from a recent study showing that ctDNA levels as measured by ddPCR during treatment with BRAF-inhibitors in patients with *BRAF*^*V600E*^ mutations do not correlate with LDH levels^22^. Our results confirm these findings and extend them to the high frequency mutational markers, ^*V600K*^*BRAF and*^*Q61H*^*NRAS*.

The ability to use ctDNA quantification from a “liquid biopsy” means that patients can have their disease status monitored more frequently and quickly without the common risks associated with a biopsy. While showing comparable accuracy and sensitivity, newly developed instrumentation means that ddPCR is a simpler methodology then the BEAMing methodology used in the BREAK-2 study^13^. Its protocols can also be readily transferred to any clinical site with the same instrumentation as used in this study. For this reason ddPCR is being adopted by numerous labs. When compared to traditional real-time PCR, due to the mutations are being quantified in absolute rather than relative counts, data can be compared more readily.

Patients who respond well to treatment can achieve undetectable ctDNA levels early while tumours are still visible on CT scan. This may be a reflection of a complete inhibition of cellular tumour activity as tumour cells respond to treatment, while tumour tissue clearance may take lengthier time. Consistently detectable ctDNA or a rising ctDNA level, may indicate persistent tumour activity and hence worse prognosis when compared to patients who have undetectable or falling ctDNA levels.

While in our and other studies^17^,^22^. *BRAF*^*V600E*^ was not detected in healthy controls, some caution must be applied when using highly sensitive methods for its presence as a diagnostic tool given the presence of *BRAF*^*V600E*^ mutations in benign nevi^23^.

In conclusion, we have shown that ctDNA as measured by ddPCR could be used not only to identify tumour-specific DNA changes but also to monitor disease progression in melanoma patients and is currently the most effective way to measure minimal residual disease.

## Methods

### Droplet digital PCR

We used the Bio-Rad QX200 ddPCR system (Bio-Rad, Hercules, CA). The ddPCR probe mastermix and primers targeting ^*V600E*^*BRAF*, ^*V600K*^*BRAF* and ^*Q61H*^*NRAS* mutations with *BRAF* and *NRAS* wild type were all purchased from Bio-Rad. The primer sequences are proprietary to the company. Data was processed using QuantaSoft v.1.6 (Bio-Rad).

Cell line DNA was used to optimise reaction conditions. We firstly tested the specificity of the method using known quantities of genomic DNA from 12 melanoma cell lines with established mutational status. Six had known ^*V600E*^*BRAF* mutations, one had ^*V600K*^*BRAF*, one had ^*G649E*^*BRA*F, two had ^*Q61H*^*NRAS*, one had ^*Q61K*^*NRAS* and 1 cell line was wild type for both *BRAF* and *NRAS*^16^. To determine the false-positive rate, 8 repeats of 10 ng and 100 ng of the wild type cell line DNA and Milli-Q water (Millipore) as no-template control were used.

Subsequently, the method was used to quantify mutant ctDNA in patient samples. To maximise the amount of DNA for each reaction, 8 μl template DNA were used per reaction. The results were compared to the patients’ clinical history, RECIST scores and LDH values.

### Patients and sample processing

This study was conducted according to the National Health & Medical Research Council (NHMRC) Australian Code for the responsible conduct of Research and the National Statement on Ethical Conduct in Human Research. Patients provided their written informed consent for the samples collected for the research study protocol, which was approved by the Human Research Ethics Committee of the Austin Hospital, Melbourne.

Four patients with stage IV melanoma and biopsy-proven ^*V600E*^*BRAF* mutation, one with ^*V600K*^*BRAF*, one with ^*Q61H*^*NRAS* and one unknown were selected based on disease status (Table 2). Blood samples were obtained when patients presented to the melanoma clinic as required based on clinical requirements. All blood samples were processed within 4 hours after collection. Plasma was isolated after centrifugation at 800 g for 10 minutes followed by a further 10 minutes centrifugation at 1600 g before it was stored at −80 ^°^C in 1 ml aliquots. Plasma DNA was extracted using the QIAamp MinElute Virus Spin Kit following manufacture’s protocol with minor modification as following; The QIAGEN Protease, buffer AL and ethanol quantities were increased 5 fold to incorporate 1 ml of plasma as the documented quantities were for 200 μl of plasma. A vacuum extraction rack with column extension tubes was used to accommodate the increased sample volume. DNA was eluted using 35 μl of Buffer AVE and quantified using the Qubit® 2.0 Fluorometer (Life Technologies).

RECIST scores were scored by the same trained medical professional on radiological scans performed appropriate for the patient judged by clinical requirement. LDH levels were analyzed by the Austin Hospital pathology department.

## Additional Information

**How to cite this article**: Chang-Hao Tsao, S. *et al*. Monitoring response to therapy in melanoma by quantifying circulating tumour DNA with droplet digital PCR for *BRAF* and *NRAS* mutations. *Sci. Rep*. **5**, 11198; doi: 10.1038/srep11198 (2015).

## Figures and Tables

**Table 1:**
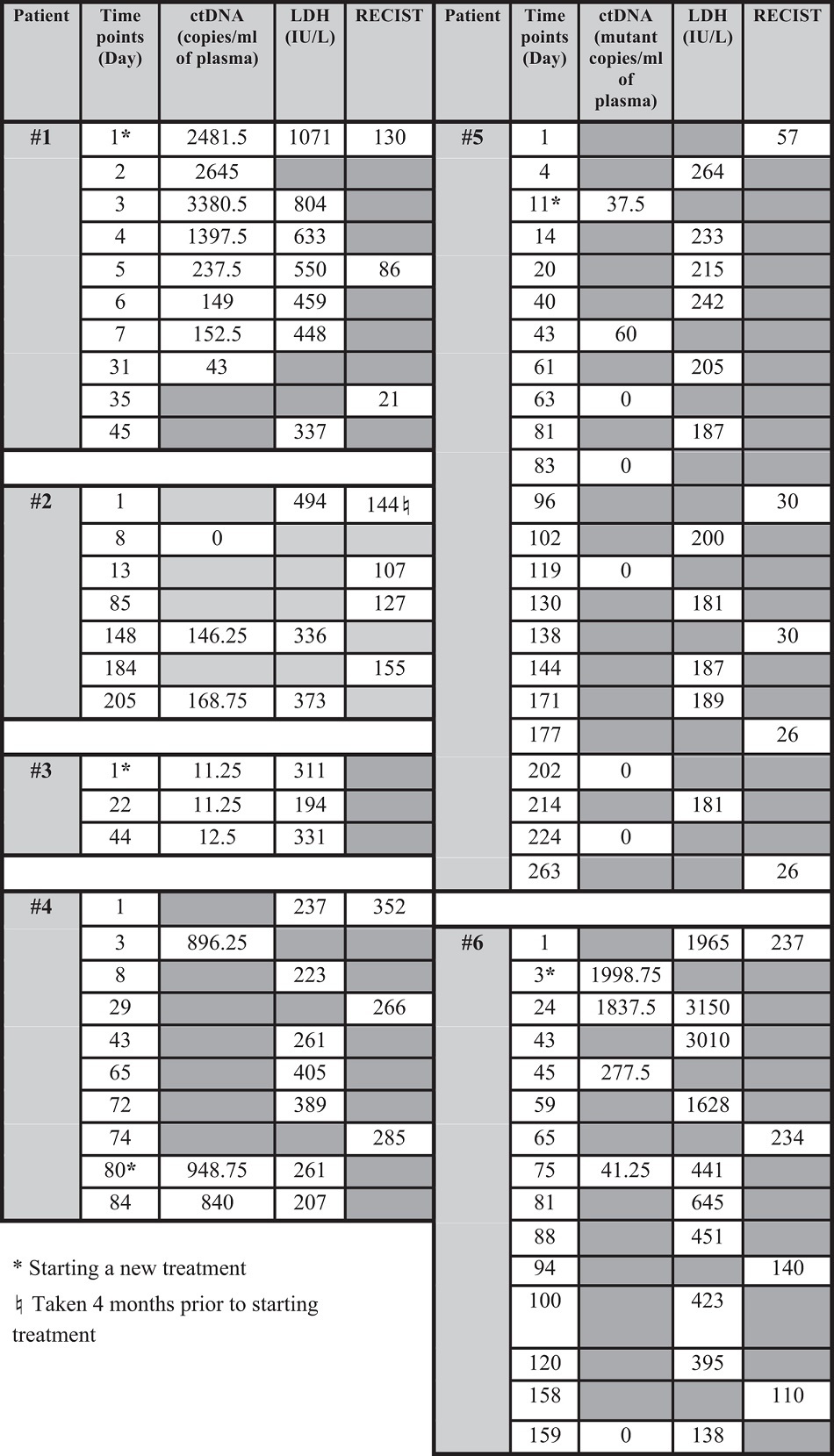
Summary of results.

**Table 2:** Patient mutation status and clinical information.

Patient	Mutation	Clinical History
1	^*V600E*^*BRAF*	Patient made drastic improvement under dabrafenib (BRAF inhibitor) treatment. Bloods were collected daily during the first week treatment.
2	^*V600E*^*BRAF*	Patient responded to dabrafenib and trametinib (MEK-inhibitor) for 4 months before disease progression.
3	^*V600E*^*BRAF*	Patient did not respond to MK3475 (PD-1 inhibitor) and continued to deteriorate clinically.
4	^*V600K*^*BRAF*	All extracranial lesions responded to MK3475 but the intracranial lesions continued to progress. Treatment was switched to ipilimumab (anti-CTLA4 antibody).
5	^*V600E*^*BRAF*	Patient was enrolled in the CheckMate 067 trial (nivolumab PD-1 inhibitor versus ipilimumab versus combination of the two) and responded to the treatment well.
6	^*Q61H*^*NRAS*	Patient was given ipilimumab and responded well with significant improvement.
